# Supplementary material for: Free and Esterified Tocopherols, Tocotrienols and Other Extractable and Non-Extractable Tocochromanol-Related Molecules: Compendium of Knowledge, Future Perspectives and Recommendations for Chromatographic Techniques, Tools, and Approaches Used for Tocochromanol Determination
Source: Molecules. 2022 Oct 4;27(19):6560. doi: 10.3390/molecules27196560 (PMC9573122; doi:10.3390/molecules27196560)
Supplement: Supplementary file 1 [file molecules-27-06560-s001.zip › molecules-1869787-supplementary.pdf]

Table S1. α-T content [ppm] and its relative amount [%] to other detected tocochromanols in the specified sources

Source	Latin name	α-T content [ppm]	α-T [%] of total tocochromanols	Other detected tocochromanols	Ref.
Potato tubers (fw)	*Solanum tuberosum*	0.63	90	β–T	[33]
Lettuce leaves (fw)	*Lactuca sativa*	3.85	55	γ–T	[33]
Spinach leaves (fw)	*Spinacia oleracea*	18.9	63	γ–T, δ–T	[33]
Arabidopsis leaves (fw)	*Arabidopsis thaliana*	18.0	90	γ–T	[33]
Sunflower seed oil	*Helianthus annuus*	672.0	96	β–T, γ–T	[33]
Sea buckthorn leaves (dw)*	*Hippophaë rhamnoides*	294.6 - 866.3	85 - 88	β–T, γ–T, PC-8	[30]
Apple leaves (dw)*	*Malus domestica*	36.3 - 130.2	58 - 88	β–T, γ–T, δ–T, α–T3, β–T3, γ–T3, δ–T3	[32]
Pear leaves (dw)*	*Pyrus communis*	53.65 - 72.91	76 - 94	β–T, γ–T, δ–T, α–T3, β–T3, γ–T3, δ–T3	[32]
Quince leaves (dw)*	*Cydonia oblonga*	74.71 - 157.73	93 - 94	β–T, γ–T, δ–T, α–T3, β–T3, γ–T3, δ–T3	[32]
Apricot leaves (dw)*	*Prunus armeniaca*	191.17 - 225.34	81 - 86	β–T, γ–T, δ–T, α–T3, β–T3, γ–T3, δ–T3	[32]
Peach leaves (dw)*	*Prunus persica*	127.2 - 203.34	88	β–T, γ–T, δ–T, α–T3, β–T3, γ–T3, δ–T3	[32]
Plum leaves (dw)*	*Prunus spinosa*	127.81 - 180.06	94 - 98	β–T, γ–T, δ–T, α–T3, β–T3, γ–T3, δ–T3	[32]
Sweet cherry leaves (dw)*	*Prunus avium*	40.26 - 124.64	88 -90	β–T, γ–T, δ–T, α–T3, β–T3, γ–T3, δ–T3	[32]
Safflower seed oil	*Carthamus tinctorius*	552.0	95	β–T, γ–T, δ–T, α–T3, γ–T3	[34]
Wild safflower seed oil	*Carthamus oxyacantha*	546.0	94	β–T, γ–T, δ–T, α–T3, γ–T3	[34]
Almonds seed oil	*Prunus dulcis*	641.1	100	–	[12]
Guelder rose seed oil	*Viburnum opulus*	1945.4	71	β–T, γ–T, δ–T	[12]
Sea buckthorn seed oil	*Hippophaë rhamnoides*	1970.2	73	β–T, γ–T, δ–T, PC-8	[12]
Milk thistle seed oil	*Silybum marianum*	605.9	87	β–T, γ–T	[12]
Wheat germ oil	*Triticum aestivum*	1917.5	64	β–T, β–T3, PC-8	[12]
Sunflower seed oil	*Helianthus annuus*	684.1	100	–	[12]
Japanese quince seed oil	*Chaenomeles japonica*	1217.9	96	β–T, γ–T, α–T3	[35]
Olive oil	*Olea europaea*	256.1	97	β–T, γ–T, δ–T, α–T3, γ–T3	[162]

** values dependent on variety, season of harvest, sex*

Table S2. β-T content [ppm] and its relative amount [%] to other detected tocochromanols in the specified sources

Source	Latin Name	β-T content [ppm]	β-T [%] of total tocochromanols	Other detected tocochromanols	Ref.
Wheat germ oil	*Triticum aestivum*	983	31	α–T, γ–T, δ–T, α–T3, γ–T3	[38]
Arabica coffee beans (dw)**	*Coffea arabica*	79.6 - 254.4	75	α–T, γ–T	[19,39]
Robusta coffee beans (dw)**	*Coffea canephora*	21 – 65.4	53 - 56	α–T, γ–T	[19,39]
Red oak acorns (dw)	*Quercus rubra*	223.7	89	α–T, γ–T, δ–T	[40]
Red oak acorn seed oil	*Quercus rubra*	785.3	94	α–T	[12]
Apple seed oil	*Malus* spp.	564.1	54	α–T, γ–T, δ–T	[41]
Dessert apple seed oil	*Malus* spp.	1242.8	55	α–T, γ–T, δ–T	[42]
Guelder rose seed oil	*Viburnum opulus*	401.4	15	α–T, γ–T, δ–T	[12]
Kirkir seed oil	*Vangueria madagascariensis*	657	59	α–T, γ–T, δ–T	[44,45]

***values depending on the degree of roasting and the country of origin*

Table S3. γ-T content [ppm] and its relative amount [%] to other detected tocochromanols in the specified sources

Source	Latin Name	γ-T content [ppm]	γ-T [%] of total tocochromanols	Other detected tocochromanols	Ref.
Flax seed oil	*Linum usitatissimum*	453.9	68	PC-8	[12]
Golden flax seed oil	*Linum flavum*	536.1	71	PC-8	[12]
Chia seed oil	*Salvia hispanica*	465.3	100	–	[12]
European beech seed oil	*Fagus sylvatica*	754	68	α–T, β–T, δ–T	[47]
Rapeseeds (dw)	*Brassica napus*	237.3	51	α–T, β–T, δ–T, PC-8	[139]
Pumpkin seed oil	*Cucurbita pepo*	674.9	100	–	[12]
Red currant seed oil	*Ribes rubrum*	1563.9	77	α–T, β–T, δ–T	[35]
Pomegranate seed oil	*Punica granatum*	3826.9	96	α–T, δ–T, α–T3, γ–T3	[35]
Watermelon seed oil	*Citrullus lanatus*	1110.1	94	α–T, β–T, δ–T, α–T3	[35]

Table S4. δ-T content [ppm] and its relative amount [%] to other detected tocochromanols in the specified sources

Source	Latin Name	δ-T content [ppm]	δ-T [%] of total tocochromanols	Other detected tocochromanols	Ref.
Borago morisiana (dw)***	*Borago morisiana*	631.7	88	α–T, γ–T	[46]
Borago officinalis (dw)***	*Borago officinalis*	380.1	86	γ–T	[46]
Borago pygmaea (dw)***	*Borago pygmaea*	857.9	77	α–T, β–T, γ–T	[46]
Borago longifolia (dw)***	*Borago longifolia*	114.7	71	γ–T	[46]
Borago trabutii (dw)***	*Borago trabutii*	420.1	82	γ–T	[46]
European beech seed oil	*Fagus sylvatica*	340.5	31	α–T, β–T, γ–T	[47]
Soybean oil	*Glycine* Willd.	332.6	24	α–T, β–T, γ–T	[12]
Guelder rose seed oil	*Viburnum opulus*	345.9	13	α–T, β–T, γ–T	[12]

**** quantified as α-T acetate equivalents*

Table S5. α-T3 content [ppm] and its relative amount [%] to other detected tocochromanols in the specified sources

Source	Latin Name	α-T3 content [ppm]	α-T3 [%] of total tocochromanols	Other detected tocochromanols	Ref.
Oat bran oil	*Avena sativa*	271	34	α–T, β–T, γ–T, δ–T, β–T3, γ–T3, δ–T3	[38]
Rye bran oil	*Secale cereale*	1604	46	α–T, β–T, γ–T, δ–T, β–T3, γ–T3, δ–T3	[38]
Cumin seed oil	*Cuminum cyminum*	699.6	85	α–T, δ–T, β–T3, γ–T3, δ–T3	[50]
Palm oil	*Elaeis guineensis*	122	23	α–T, β–T, γ–T, δ–T, β–T3, γ–T3, δ–T3	[125]

Table S6. β-T3 content [ppm] and its relative amount [%] to other detected tocochromanols in the specified sources

Source	Latin Name	β-T3 content [ppm]	β-T3 [%] of total tocochromanols	Other detected tocochromanols	Ref.
Spelt bran oil	*Triticum spelta*	2086	60	α–T, β–T, α–T3, γ–T3, δ–T3	[38]
Wheat bran oil	*Triticum aestivum*	1635	49	α–T, β–T, γ–T, δ–T, α–T3, γ–T3, δ–T3	[38]
Nigella seed oil	*Nigella sativa*	1195.1	75	α–T, β–T, γ–T, δ–T, α–T3, δ–T3	[51]
Rye bran (dw)	*Oryza sativa*	37	35	α–T, β–T, γ–T, δ–T, α–T3	[158]
Wheat bran (dw)	*Triticum aestivum*	74	58	α–T, β–T, γ–T, δ–T, α–T3	[158]
Spelt bran (dw)	*Triticum spelta*	109	69	α–T, β–T, γ–T, δ–T, α–T3	[158]

Table S7. γ-T3 content [ppm] and its relative amount [%] to other detected tocochromanols in the specified sources

Source	Latin Name	γ-T3 content [ppm]	γ-T3 [%] of total tocochromanols	Other detected tocochromanols	Ref.
Latex from rubber tree	*Hevea brasiliensis*	260	83	α–T3, δ–T3	[53]
Annatto seed oil	*Bixa orellana*	20000	12	δ–T3	[54]
Grape-seed oil	*Vitis vinifera*	1575	72	α–T, γ–T, δ–T, α–T3, β–T3, δ–T3	[57]
Washingtonia filifera seed oil	*Washingtonia filifera*	963.3	72	α–T, β–T, γ–T, δ–T, α–T3, δ–T3	[55]
Cranberry seed oil	*Vaccinium macrocarpon*	1800	92	γ–T, α–T3, δ–T3	[56]
Arctic cranberry seed oil	*Vaccinium oxycoccos*	1900	95	γ–T, α–T3	[56]
Lingonberry seed oil	*Vaccinium vitis-idaea*	1200	85	α–T3	[56]
Coriander seed oil	*Coriandrum sativum*	302.6	75	α–T, γ–T, δ–T, α–T3, δ–T3	[50]
Parsley seed oil	*Petroselinum sativum*	175.2	75	γ–T, α–T3, δ–T3	[50]
Celery seed oil	*Apium graveolens*	608.9	83	α–T, α–T3, δ–T3	[50]
Dill seed oil	*Anethum graveolens*	668.5	76	α–T, δ–T, α–T3, β–T3, δ–T3	[50]
Carrot seed oil	*Daucus carota*	228.2	57	α–T, α–T3, β–T3, δ–T3	[50]
Caraway seed oil	*Carum carvi*	1183.9	76	γ–T, δ–T, α–T3, δ–T3	[50]
Fennel seed oil	*Foeniculum vulgare*	393.2	56	β–T, γ–T, α–T3, β–T3, δ–T3	[50]

Table S8. δ-T3 content [ppm] and its relative amount [%] to other detected tocochromanols in the specified sources

Source	Latin Name	δ-T3 content [ppm]	δ-T3 [%] of total tocochromanols	Other detected tocochromanols	Ref.
Annatto seed oil	*Bixa orellana*	149000	88	γ–T3	[54]
Latex from rubber tree	*Hevea brasiliensis*	52	17	α–T3, γ–T3	[53]
Giant hogweed seed oil	*Heracleum mantegazzianum*	360.1	32	α–T3, β–T3, δ–T3	[50]
Lychee seed oil	*Litchi chinensis*	7675	82	α–T, β–T, γ–T, δ–T, α–T3, γ–T3	[63]

Table S9. PC-8 content [ppm] and its relative amount [%] to other detected tocochromanols in the specified sources

Source	Latin Name	PC-8 content [ppm]	PC-8 [%] of total tocochromanols	Other detected tocochromanols	Ref.
Cecropia sp. leaves (dw)	*Cecropia* sp.	1783	51	unpublished	[65]
Pseudobombax munguba leaves (dw)	*Pseudobombax munguba*	3640	64	unpublished	[65]
Tabaernamontana siphilitica leaves (dw)	*Tabaernamontana siphilitica*	276	44	unpublished	[65]
Apeiba sp. leaves (dw)	*Apeiba* sp.	617	46	unpublished	[65]
Corchorus olitorius seed oil	*Corchorus olitorius*	109	6	α–T, β–T, γ–T, δ–T	[63]
Erythrophleum fordii seed oil	*Erythrophleum fordii*	167	16	α–T, β–T, γ–T, δ–T, α–T3	[63]
Connarus paniculatus seed oil	*Connarus paniculatus*	76	14	α–T, β–T, γ–T	[63]
Rapeseeds (dw)	*Brassica napus*	67.2	12	α–T, β–T, γ–T, δ–T	[138]
Flaxseed oil	*Linum usitatissimum*	216	23	α–T, γ–T, δ–T	[12]

Table S10. Summary of applied NPLC methods for the determination of tocochromanol related compounds

Column	Mobile phase	Temperature, Flow rate, Gradient or isocratic, Run time	Analytes	Matrix	Detector	Ref.
Kromasil Phenomenex (250 x 4.6mm; 5 μm)	hx:EtOAc:AcOH (97.3:1.8:0.9)	—; 1.6mL/min; Isocratic; 25 min	4Ts, 4T3s	Cereals	FLD	[11]
LiChrosorb Si60 (250 x 4.6 mm; 5 μm)	hx:dioxane (96:4)	20 °C; 1mL/min; Isocratic; 40 min	4Ts, 4T3s, PC-8	Flaxseed oil	FLD	[13]
Lichrosphere Si-60 Merck (250 x 4.0 mm; 5 μm)	hx:IPA (99:1)	—; 1mL/min; Isocratic; 15 min	αT, βT, γT	Coffee oil	FLD	[18]
LiChrosorb Si 60 (250 x 4.6 mm; 5 μm)	hx:dioxane (97:3)	—; 1.5mL/min; Isocratic; —	4Ts	Coffee beans	FLD	[19]
Alltima SI column (250 x 4.6 mm; 5 μm)	hx:EtOAc:AcOH (97.3:1.8:0.9)	20 °C; 1.6mL/min; Isocratic; —	4Ts	Lupinus mutabilis sweet seeds	FLD	[21]
Supelcosil LC-Si (250 x 4.6 mm; 5 μm)	EtOAc:AcOH:hx (or hp) (1:1 :198)	—; 1.5mL/min; Isocratic; (if hex: 18 min; if hep: 21 min)	4Ts, 4T3s	Wheat, oat, rye dough and bread	FLD	[22]
Silica (300 mm × 3.9 mm; 5 μm)	hx:IPA (99.8:0.2)	—; 1.3mL/min; Isocratic; —	α-T, γ-T3, δ-T, δ-T3, desmethyl-T3, didesmethyl-T3	Extracts of stabilized rice bran	FLD	[26]
Hypersil silica column (200 x 4.6 mm; μm)	hx:dioxane (96:4)	40 °C; 1mL/min; Isocratic; 20 min	4Ts, 4T3s, α-TAc	STD, crude palm oil	UV	[29]
LiChrosorb Si 60 (250 x 4.6 mm; 5μm)	hx:dioxane (97:3)	20 °C; 1.5mL/min; Isocratic; 20 min	αT, βT, γT, PC-8	Leaves of Sea Buckthorn (H. rhamnoides L.)	FLD	[30]
SupelcosilTM LC-SI (75 x 3.0 mm; 3 μm)	hx:dioxane (98:2)	21 °C; 0.7mL/min; Isocratic; 8 min	4Ts, tocol (IS)	Coffee beans	DAD FLD	[39]
Diol (250 x 4.6 mm; μm)	hp:MTBE (99:1)	—; 1.3mL/min; Isocratic; —	4Ts, 4T3s	Sunflower oil	FLD	[51]
Phenomenex Luna Silica (250 x 4.6 mm; 5 μm)	hp:EtOAc (93:7)	30 °C; 0.6mL/min; Isocratic; —	4T3s, αT, 11'-αT1, 7,11'-αT2	T3 rich fraction	FLD	[52]
Particil Pac (250 x 4.6 mm; 5 μm)	hx:THF (94:6)	—; 1mL/min; Isocratic; —	4Ts, 4T3s	Grape seeds	FLD DAD	[60]
Diol (250 x 4.6 mm)	hp:MTBE (99:1)	—; 1.3mL/min; Isocratic; —	4Ts, 4T3s, PC-8	Different oils	FLD	[63]

Column	Mobile phase	Temperature, Flow rate, Gradient or isocratic, Run time	Analytes	Matrix	Detector	Ref.
Zorbax SIL<br>(250 x 4.6 mm; 5 μm)	hx:dioxane:IPA<br>(985:10:5)	30 °C; 1.5mL/min; Isocratic; —	4Ts, 4T3s, αT1	Palm, rice bran oil	FLD	[69]
Extrasil Si<br>(250 x 4.6 mm; 3 μm)	hx:MTBE<br>(97:3)	—; 1.5mL/min; Isocratic; —	γT1, βT1	Leaves of Kalanchoe daigremontiana and Phaseolus coccineus	FLD<br>APPI-MS	[74]
Nucleodur 100 Si<br>(250 x 4 mm; 5 μm)	hx:IPA<br>(98.8:1.2)	—; 1mL/min; Isocratic; —	α-tocopherol quinone, αT	Vegetable Oils	DAD	[88]
Reprosil 100 C18<br>(250 x 4 mm; 5 μm)<br>Nucleosil 125C 18<br>(125 x 4 mm; 5 μm)	MeOH:IPA<br>(65:35)	—; 1mL/min; Isocratic; —	α-tocopherol esters (oleic, palmitic, linolenic)			
LiChrosorb Si 60 Hibar RT<br>(250 x 4 mm; 5 μm)	hx:IPA<br>(99.15:0.85)	—; 1mL/min; Isocratic; —	4Ts, 4T3s	Distillates of structured lipids	FLD	[89]
Inertsil silica<br>(250 x 4.6 mm; μm)	hx:dioxane<br>(97:3)	30 °C; 2mL/min; Isocratic; 20 min	4Ts, 4T3s	Cereals	FLD	[102]
Kromasil Phenomenex Si<br>(250 x 4.6 mm; 5 μm)	hx:EtOAc:AcOH<br>(97.3:1.8:0.9)	—; 1.6mL/min; Isocratic; 25 min	4Ts, 4T3s	Cereals	FLD	[108]
LiChrospher Si60<br>(250 x 4 mm; 5 μm)	hx:dioxane<br>(96:4)	—; 2mL/min; Isocratic; —	αT, γT	Roasted pumpkin seeds	FLD	[121]
LiChrosorb Si 60<br>(250 x 4.6 mm; 5 μm)	hx:dioxane<br>(96:4)	—; 1mL/min; Isocratic; —	4Ts, PC-8	Rapeseed oil	FLD	[122]
Luna NH2 100<br>(250 x 4.6 mm; 5 μm)	hx:IPA<br>(96:4)	—; 2mL/min; Isocratic; —	4Ts	Canola seed oil	FLD	[123]
ProntoSil 120-5 Diol<br>(250 x 4.0 mm; 5 μm)	A: hx<br>B: dioxane<br>C: MTBE	—; 1mL/min; Gradient; 60 min	4Ts, 4T3s, and oxidation products	STD	DAD<br>FLD<br>EI-MS	[132]
Alltima SI 5U (Alltech)<br>(250 x 4.6 mm; 5 μm)	hx:dioxane<br>(96:4)	—; 2mL/min; Isocratic; 15 min	4Ts, 4T3s	STD	FLD	[134]
Inertsil SI<br>(250 x 4.6 mm; 5 μm)	hx:dioxane<br>(95:5)	—; 2mL/min; Isocratic; 15 min				

Column	Mobile phase	Temperature, Flow rate, Gradient or isocratic, Run time	Analytes	Matrix	Detector	Ref.
Genesis silica (250 x 4.6 mm; 4 μm)	hx:dioxane (96:4)	—; 1.5mL/min; Isocratic; 22 min	4Ts, 4T3s	STD	FLD	[134]
LiChrosorb Diol Hibar (250 x 4 mm; 5 μm)	hx:MTBE (96:4)	—; 2mL/min; Isocratic; 30 min				
Hypersil APS-2 (250 x 4.6 mm; 5 μm)	hx:dioxane (95:5)	—; 2.5mL/min; Isocratic; 16 min				
LiChrosorb NH2 (250 x 4 mm; 5 μm)	hx:MTBE:THF:MeOH (79:20:1:0.1)	—; 1mL/min; Isocratic; 18 min				
Waters p-Bondapak NH2 (300 x 3.9 mm; 10 μm)	cyclohexane:MTBE (90:10)	—; 1mL/min; Isocratic; 20 min	αT, 5,7-dimethyltocol (IS), βT,γT,δT	STD	FLD	[135]
Alltech Lichrosorb DIOL (250 x 4.6 mm; 10 μm)	hx:MTBE (90:10)	—; 1mL/min; Isocratic; 32 min				
ES Industries Chromega Diol (250 x 4.6 mm; 5 μm)	hx:diisopropyl ether (90:10)	—; 1mL/min; Isocratic; 32 min				
LiChrospher 100 diol (250 x 4 mm; 5 μm)	A:hx B:MTBE	—; 1.3mL/min; Gradient; 72 min	αTAc, 4Ts, 4T3s, PC-8	Infant formulas, human milk, breakfast cereals, multivitamin juices, isotonic beverages	FLD	[136]
Diol (250 x 4.6 mm)	hp:MTBE (99:1)	—; 1.3mL/min; Isocratic; —	4Ts, 4T3s, PC-8	Citrus seed oil	FLD	[138]
LiChrosorb Si 60 (250 x 4.6 mm; 5 μm)	hx:dioxane (97:3)	—; 1.5mL/min; Isocratic; —	4Ts, PC-8	Rapeseed oil	FLD	[139]
Zorbax SIL (250 x 4.6 mm)	hx:IPA (99:1)	room temperature; 2mL/min; Isocratic; 6 min	4Ts, αT3, γT3, δT3	STD	UV-VIS	[145]
Inertsil 5 SI (250 x 3 mm; μm)	dioxane:hx (3.5:96.5)	room temperature °C; 0.7mL/min; Isocratic; 25 min	4Ts and 4T3s	Olive oil	DAD FLD ELSD	[162]

**Column**	**Mobile phase**	**Temperature, Flow rate, Gradient or isocratic, Run time**	**Analytes**	**Matrix**	**Detector**	**Ref.**
Varian A2014250X020 propyl-amine (250 x 2 mm; 3 μm)	(A) hx<br>(B) hx:dioxane 1:1	—; 0.3-0.5mL/min; Gradient; 15 min	4Ts, 4T3s, α-tocopherylquinone, 2H6-R-tocopherol	Human plasma	APCI(+)-QqQ	[192]
Phenomenex Luna Silica (250 x 4.6 mm; 5 μm)	hp:THF (1000:40)	—; 1mL/min; Isocratic; 30 min	4Ts, 4T3s, 11'-αT1, 11'-γT1	Pumpkin seeds	FLD	[193]
Zorbax Polaris Silica (150 x 4.6 mm; 3 μm)	(A) hp<br>(B) EtOAc	40 °C; 2mL/min; Gradient; 15 min	4Ts, 4T3s, 11'-αT1	Palm-derived T3 rich fraction	PDA	[194]

Table S11. Summary of applied RPLC methods for the determination of tocochromanol related compounds

Column	Mobile phase (*v/v*)	Temperature, Flow rate, Gradient or isocratic, Run time	Analytes	Matrix	Detector	Ref.
Kinetex PFP (100 x 4.6 mm; 2.6 μm)	MeOH:H_2O (76:24)	40 °C; 1.2 mL/min; Isocratic; 90 min	4Ts, 4T3s, αT1	Cyanobacteria, microalgae	FLD	[15]
	A:MeOH:H_2O (80:20)<br>B:MeOH:H_2O (97:3)	40 °C; 0.6 mL/min; Gradient; 16 min			DAD<br>APCI-Orbitrap	
Nucleosil 100 C18 (250 x 4 mm; 5 μm)	ACN:MeOH:H_2O (72:8:1)	—; 1.5 mL/min; Isocratic; —	αT, γT, δT	Young leaves of runner bean	FLD	[31]
YMC C30 (250 x 4.6 mm; 3 μm)	MeOH (25 mM sodium chlorate, 2 mM $HClO_4$)	—; 1.5 mL/min; Isocratic; 11.8 min	nitro-γT		ECD	
Nucleosil 100 C18 (250 x 4 mm; 5 μm)	MeOH:hx (85:15)	24 °C; 1.5 mL/min; Isocratic; 10 min	PC-8	Plant Oils	FLD	[36]
	ACN:MeOH:H_2O (72:8:1)	24 °C; 2 mL/min; Isocratic; 12 min	αT, (βT+γT), δT, αT3, (βT3+γT3), δT3			
YMC C30 (250 x 4.6 mm, 3 μm)	ACN:MeOH:H_2O (72:8:1)	24 °C; 1 mL/min; Isocratic; —	βT3, γT3	Plant oils	FLD	[36]
Nucleosil 100 C18 (250 x 4 mm; 5 μm)	ACN:MeOH:H_2O (72:8:1)	—; 1.5 mL/min; Isocratic; 25 min	αT, (βT+γT), δT, αT3, (βT3+γT3), δT3	Oils	FLD	[37]
	MeOH:hx (340:20)	—; 1.5 mL/min; Isocratic; 25 min	PC-8			

Column	Mobile phase (*v*/*v*)	Temperature, Flow rate, Gradient or isocratic, Run time	Analytes	Matrix	Detector	Ref.
YMC C30 (150 x 3.0 mm; 3 μm)	A: MeOH:H_2O (96:4) B: MeOH:MTBE:H_2O (4:92:4)	25 °C; 0.42 mL/min; Gradient; —	4Ts	Seed oils	DAD	[41]
Luna PFP (150 x 4.6 mm; 3 μm)	MeOH:H_2O (93:7)	40 °C; 1 mL/min; Isocratic; 13 min	4Ts	Seed oils	FLD	[42]
C30 phase (250 x 4.6 mm, 5 μm)	ACN:MeOH:dichlormethane (72:22:6)	30 °C; 1 mL/min; Isocratic; —	4Ts	Viburnum opulus pomace and berries	FLD	[43]
Brownlee C18 (100 x 4.6 mm; 5 μm)	MeOH:ACN:H_2O (50:44:6)	—; 1 mL/min; Isocratic; —	αT, (βT+γT), δT, αT3, (βT3+γT3), δT3	Berries seed oils	DAD	[56]
Nucleosil 100 (250 x 4 mm; 5 μm)	MeOH:hx (340:20)	—; 1.5 mL/min; Isocratic; 15 min	γT, αT, plastoquinol-9, PC-8	Leaves of Arabidopsis	DAD FLD	[68]
Intersil ODS-2 (250 x 4.6 mm; 5 μm)	MeOH:H_2O (95:5)	30 °C; 1 mL/min; Isocratic; 35 min	αT, (βT + γT), δT, αT3, (βT3 + γT3), δT3, αT1	Palm, rice bran oil	FLD	[69]
Supelcosil LC18 (250 x 4.6 mm; 5 μm)	50 mM sodium perchlorate in MeOH	—; 1 mL/min; Isocratic; 15 min	MDT, α-T	Eggs of the Pacific Salmon	ED	[70]
2 x Supelcosil LC18 (250 x 4.6 mm; 5 μm)	50 mM sodium perchlorate in MeOH:H_2O (50:1)	—; 1 mL/min; Isocratic; 50 min	MDT, αT	Marine organisms	ECD	[71]
Develosil RP C30 (250 x 4.6 mm)	MeOH	—; 1 mL/min; isocratic; —	4Ts, 4T3s, αT1	Palm oil	FLD	[72]
Nucleosil 100 C18 (250 x 4 mm; 5 μm)	ACN:MeOH:H_2O (72:8:1)	—; 1.5 mL/min; Isocratic; —	δT1, (γT1+βT1), δT, γT, αT	Leaves of Kalanchoe daigremontiana and Phaseolus coccineus	FLD APPI-MS	[74]
Kinetex PFP (150 x 3.0 mm; 2.6 μm)	A:MeOH:H_2O (85:15) B:MTBE:MeOH:H_2O (80:18:2)	24 °C; 0.3 mL/min; Gradient; 45 min	4Ts, 4T3s	Cooked and raw vegetables	FLD	[101]
Develosil RP Aqueous (C30) (150 x 3.0 mm; 3 μm)	A:MeOH:H_2O (91:9) B:MTBE:MeOH:H_2O (80:18:2)	18 °C; 0.5 mL/min; Gradient; 63 min				

Column	Mobile phase (*v*/*v*)	Temperature, Flow rate, Gradient or isocratic, Run time	Analytes	Matrix	Detector	Ref.
DOCOSIL-B C22 (250 x 4.6 mm; 2.6 μm)	MeOH	—; 1 mL/min; Isocratic; 45 min	δT, γT, αT, MDT, 11'-α-T1	Tuna oil deodorization Scum	FLD	[130]
Develosil RPC30 (250 x 4.6 mm; 5 μm)	MeOH:H_2O (99:1)	10 °C; 1 mL/min; Isocratic; 37 min	4Ts, tocol (IS)	Oils	FLD	[141]
YMC C30 (250 x 4.6 mm; 5 μm)	MeOH:H_2O (95:5)	5 °C; 1 mL/min; Isocratic; 30 min				
Cosmosil π-NAP (250 x 4.6 mm; 5 μm)	MeOH:H_2O (90:10)	30 °C; 1.5 mL/min; Isocratic; 15 min			DAD + FLD	[141]
Phenomenex Kinetex PFP (150 x 4.6 mm; 2.6 μm)	A: MeOH:H_2O B:MeOH (80:20)	40 °C; 0.8 mL/min; Gradient; 60 min	4Ts, 4T3s, α-T1	Palm fruits	FLD	[142]
Nucleosil 100 C18 (250 x 4 mm; 5 μm)	A:MeOH B: MeOH:IPA:ACN (40:50:10)	25 °C; 1-1.2 mL/min; Gradient; 26 min	αT, (βT+γT), δT, αT3, (βT3+γT3), δT3	Cereals	FLD	[146]
PerfectSil Target ODS-3 (250 x 4.6 mm; 3 μm)	A: IPA B: H_2O	7 °C; 0.24-0.31 mL/min; Gradient; 62 min	4Ts, 4T3s	Cereals	FLD	[147]
COSMOSIL π-NAP (250 x 4.6 mm; 5 μm)	MeOH:H_2O (90:10)	—; 1 mL/min; Isocratic; 20 min	4Ts	Edible oils	DAD	[148]
Cosmosil 5PYE (250 x 4.6 mm; μm)	MeOH:H_2O (95:5)	30 °C; 1 mL/min; Isocratic; —	αT, βT, γT	Hazelnuts	DAD	[149]
Quasar SPP RP-Amide (150 x 4.6 mm; 2.6 μm)	MeOH:H_2O (1% FA) (9:91)	40 °C; 1 mL/min; Isocratic; 30 min	4Ts, 4T3s	STD	FLD	[150]
Taxsil PFP (250 x 4.6 mm; 5 μm)	MeOH:H_2O (92:8)	—; 1 mL/min; Isocratic; 20 min	4Ts	STD, soybean oil and deodorizer distillate, vitamin E capsule	DAD	[151]
Acquity BEH C18 (50 x 2.1 mm; 1.7 μm)	A: MeOH:H_2O (90:10) B: MeOH	60 °C; 0.8 mL/min; Gradient; —	αT, phylloquinone, PC-8, plastoquinone-9	Plant tissues	APCI-QTOF	[153]
Eclipse XDB C18 (150 x 4.6 mm; 5 μm)	ACN:MeOH:IPA (40:55:5)	30 °C; 0.8 mL/min; Isocratic; 13 min	αT, (βT+γT), δT, αT3, (βT3+γT3), δT3	Barley	FLD	[154]

Column	Mobile phase (*v/v*)	Temperature, Flow rate, Gradient or isocratic, Run time	Analytes	Matrix	Detector	Ref.
HyPurity C18 (250 x 4.6 mm; 5 µm)	THF:MeOH (10:90)	21 °C; 1 mL/min; Isocratic; 10 min	αT, (βT+γT), δT	Olive oil	DAD FLD	[159]
Altech C18 (250 x 4 mm; 5 µm)	MeOH:1,4 dioxane:H_2O +75 mM $NaClO_4$ (86:10:4)	—; 1 mL/min; Isocratic; —	4Ts, 4T3s	Cereals	ECD	[160]
Superspher 100 RP18 (250 x 4 mm; 4 µm)	EtOH:MeOH (2.5 mM $HClO_4$, 7.5 mM $NaClO_4$) (90:10)	12.5 °C; 0.6 mL/min; Isocratic; —	4Ts	Human plasma	ECD	[161]
LiChrosorb RP18 (120 x 4.6 mm; 5 µm)	MeOH-H_2O (98:2)	—; 1.5 mL/min; Isocratic; 8 min	αT, γT, δT, αT-Ac	Cultured endothelial cells, human platelets	UV FLD ELSD	[164]
Waters RP8 (150 x 2.1 mm; 3.5 µm)	A:H_2O(0.1% FA) B:ACN (0.1 % FA)	room temperature; 0.3 mL/min; Gradient; —	αT, αT quinone	Human plasma	ESI-MS/MS	[165]
XTerra MS C18 (100 x 2.1 mm; 3.5 µm)	6 mM ammonia in MeOH:H_2O (97:3)	25 °C; 0.2 mL/min; Isocratic; —	αT, γT, δT, αT3, γT3, δT3	Cereals	LC-ESI-TOF	[187]
In-house prepared C30 (250 x 4.6 mm; 3 µm)	A: Acetone B: H_2O	—; —; Gradient; —	4Ts, αT-Ac	Vegetable extracts, vitamin supplements	ESI-MS	[188]
Hypersil Fluophase (PFP) (200 x 4.6 mm; 5 µm)	MeOH:H_2O (95:5)	23 °C; 0.5 mL/min; Isocratic; 15 min	4Ts	Sunflower oil, milk	ESI-MS APCI-MS	[189]
Ascentiss Express F5 (PFP) (150 x 4.6 mm; 5 µm)	MeOH:H_2O (85:15)	—; 1 mL/min; Isocratic; —	4Ts, 4T3s	Fruit and vegetable foods	FLD APCI-MS	[191]

**"—" – not provided, 4Ts or 4T3s – in this study all four tocopher-ol/tocotrienol homologues (α, β, γ, δ) were analytes/separated, ACN – acetonitrile, APCI – atmospheric pressure chemical ionization, DAD – diode array detector, dioxane – 1,4-dioxane, ECD – electrochemical detector, ELSD – evaporative light scattering detector, ESI - elec-trospray ionization, EtOH – Ethanol, FA – formic acid, FLD – fluorescence detector, hx – hexane, IPA - 2-propanol, IS – internal standard, MS – mass spectrometry, MeOH – Methanol, NH4OAc – ammonium acetate, TOF – time-of-flight.*

Table S12. Summary of applied SFC methods for the determination of tocochromanol related compounds

Column	Mobile phase	Temperature, Flow rate, BPR, Gradient or isocratic, Run time	Analytes	Matrix	Detector	Ref.
Halo Biphenyl (250 x 4.6 mm; 2.7 µm)	(A) CO2 (B) MeOH	25 °C; 2 mL/min; 10 MPa; Gradient; 15 min	4Ts, 4T3s, PC-8	Cold-pressed oils	DAD	[12]

Column	Mobile phase	Temperature, Flow rate, BPR, Gradient or isocratic, Run time	Analytes	Matrix	Detector	Ref.
Acquity UPC2 BEH (100 x 3.0 mm; 1.7 µm)	A:CO2 B:MeOH (99.5:0.5 v/v)	50 °C; 2.5 mL/min; 12.41 MPa; Isocratic; 5.5 min	4Ts, 4T3s, 11'-α-T1, 7,11'-αT2	T3 rich fraction	DAD APCI-MS	[52]
Amine Luna NH2 (100 x 3.0 mm; 1.7 µm)	A:CO2 B:EtOH (FA 0.1 %)	30 °C; 1.5 mL/min; 13 MPa; Gradient; 5 min	α-TAc, 4Ts, 4T3s	Soybean oil	DAD ESI-MS APCI-MS APPI-MS	[94]
Kinetex C18 (100 x 3.0 mm; 1.7 µm)	A: CO2 B: MeOH (99.8:0.2)	40 °C; 4.5 mL/min; 15 MPa; Isocratic; 2 min	4Ts	Fruit seeds	DAD	[144]
Acquity UPC2 HSS C18 SB (100 x 3.0 mm; 1.7 µm)	A: CO2 B: MeOH	35 °C; 2.3 mL/min; 12.41 MPa; Gradient; 13 min	4 Ts	Hemp and waste fish oil	PDA	[181]
Acquity UPC2 BEH 2-EP (100 x 3.0 mm; 1.7 µm)	A: CO2 B:MeOH:IPA (1:1) Gradient elution	50 °C; 1.5 mL/min; 12.41 MPa; Gradient; 6.2 min	4Ts, 4T3s	Moringa oleifera leaves	DAD	[183]
Spherisorb Si (150 x 4.6 mm; 5 µm)	A:CO2 B:MeOH (1 M NH4OAc) (98:2)	—; 1.5 mL/min; 15 MPa; Isocratic; 8 min	4Ts, 5,7,8-trimethyl-6-hydroxy-chromanol (IS)	Vegetable oils	ECD	[185]
Inertsil CN-3 (250 x 4.6 mm; 5 µm)	A:CO2 B:MeOH (99:1) C: (make-up pump): 1.0 M NH_4OAc	40 °C; (A+B=3 mL/min; C: 0.5 mL/min); 15 MPa; Isocratic; 30 min	4Ts, 4T3s, 5,7,8-trimethyl-6-hydroxy-chromanol (IS)	Nutrition supplements	ECD	[186]

** "—" – not provided, 4Ts or 4T3s – in this study all four tocopher-ol/tocotrienol homologues (α, β, γ, δ) were analytes/separated, APCI – atmospheric pressure chemical ionization, APPI – atmospheric pressure photoionization, BPR – back-pressure regulator, DAD – diode array detector, ECD – electrochemical detector, ESI - electrospray ionization, EtOH – Ethanol, IPA - 2-propanol, IS – internal standard, MeOH – Methanol, MS – mass spectrometry, NH4OAc – ammonium acetate*
